# Fleas of Small Mammals on Reunion Island: Diversity, Distribution and Epidemiological Consequences

**DOI:** 10.1371/journal.pntd.0003129

**Published:** 2014-09-04

**Authors:** Vanina Guernier, Erwan Lagadec, Gildas LeMinter, Séverine Licciardi, Elsa Balleydier, Frédéric Pagès, Anne Laudisoit, Koussay Dellagi, Pablo Tortosa

**Affiliations:** 1 Centre de Recherche et de Veille sur les Maladies Emergentes dans l'Océan Indien, Plateforme de Recherche CYROI, Sainte Clotilde, Reunion Island, France; 2 Institut de Recherche pour le Développement, Sainte Clotilde, Reunion Island, France; 3 Unité Mixte de Recherche Contrôle des Maladies Animales Exotiques Emergentes, CIRAD, Montpellier, France; 4 Regional Office of the French Institute for Public Health Surveillance (Cire OI - Institut de veille sanitaire), Saint Denis, Reunion Island, France; 5 Institute of Integrative Biology, School of Biological Sciences, University of Liverpool, Liverpool, United Kingdom; 6 Evolutionary Ecology Group, University of Antwerp, Antwerp, Belgium; 7 Université de La Réunion, joint chair CNRS-Université de La Réunion, Sainte Clotilde, Reunion Island, France; University of Tennessee, United States of America

## Abstract

The diversity and geographical distribution of fleas parasitizing small mammals have been poorly investigated on Indian Ocean islands with the exception of Madagascar where endemic plague has stimulated extensive research on these arthropod vectors. In the context of an emerging flea-borne murine typhus outbreak that occurred recently in Reunion Island, we explored fleas' diversity, distribution and host specificity on Reunion Island. Small mammal hosts belonging to five introduced species were trapped from November 2012 to November 2013 along two altitudinal transects, one on the windward eastern and one on the leeward western sides of the island. A total of 960 animals were trapped, and 286 fleas were morphologically and molecularly identified. Four species were reported: (i) two cosmopolitan *Xenopsylla* species which appeared by far as the prominent species, *X. cheopis* and *X. brasiliensis*; (ii) fewer fleas belonging to *Echidnophaga gallinacea* and *Leptopsylla segnis*. *Rattus rattus* was found to be the most abundant host species in our sample, and also the most parasitized host, predominantly by *X. cheopis*. A marked decrease in flea abundance was observed during the cool-dry season, which indicates seasonal fluctuation in infestation. Importantly, our data reveal that flea abundance was strongly biased on the island, with 81% of all collected fleas coming from the western dry side and no *Xenopsylla* flea collected on almost four hundred rodents trapped along the windward humid eastern side. The possible consequences of this sharp spatio-temporal pattern are discussed in terms of flea-borne disease risks in Reunion Island, particularly with regard to plague and the currently emerging murine typhus outbreak.

## Author Summary

Fleas are blood-feeding parasites involved in the transmission of several arthropod borne pathogens. Rat-fleas (*Xenopsylla* spp.) are known vectors of bubonic plague together with other human diseases receiving less attention such as murine typhus. This latter disease was recorded for the first time in 2011 on Reunion Island where seven human cases were further confirmed within the following year. The outbreak motivated a large survey of fleas, as these insects of major veterinary and medical importance have never been investigated on this oceanic island. We collected fleas on almost 1000 small wild mammals trapped on two altitudinal transects along the humid eastern and dry western sides of the island. Our data reveal the presence of four cosmopolitan flea species and shows an astonishing distribution pattern: 81% of all collected fleas were sampled on the western transect while not a single rat-flea was sampled on the eastern humid side of the island. Interestingly, this distribution did at least in part overlay the map of murine typhus human cases. These data stimulate the need for a diagnosis of pathogens in natural flea populations together with a comprehensive distribution map of fleas, allowing a risk assessment of flea-borne diseases in humans.

## Introduction

Reunion is a small oceanic island of volcanic origin located in the Indian Ocean, Southern Hemisphere (21°6′S and 55°36′E) that forms, together with Mauritius and Rodrigues Islands, the Mascarene archipelago. This oceanic island is geographically isolated from continental landmasses and located within one of the 34 recognized world biodiversity hotspots [1]. The island lies therefore in a biogeographic context favourable to species radiation and potentially high endemism. Its dramatic relief has shaped a highly contrasted climate: the mountainous centre (>3,000 meters) separates a humid windward coast (scoring some rain world records) from a dry leeward coast, which lower part consists mainly in savannah. This peculiar situation has led to the evolution of a strong vegetal endemism with a well-described altitudinal succession of vegetal species observed on both windward and leeward coasts [2]. The diversity of terrestrial animals, specifically mammals, is clearly much less prominent: the only endemic mammal species is the insectivorous bat *Mormopterus francoimoutoui* [3]. Following human colonization which started in the XVII^th^ century, five small mammal species have been introduced, namely the insectivores *Suncus murinus* Linnaeus 1766 (Asiatic house shrew) and *Tenrec eucaudatus* Schreber 1778 (tailless tenrec) from Madagascar, and the three cosmopolitan murid rodents *Rattus rattus* Linnaeus 1758 (black rat), *Rattus norvegicus* Linnaeus 1769 (brown rat) and *Mus musculus* Linnaeus 1758 (house mouse). Tropical countries and especially tropical islands are known at higher risk for the emergence or re-emergence of infectious diseases [4]. Therefore, updated information on zoonotic pathogens and on the diversity and distribution of their arthropod vectors is warranted for a quicker response to outbreaks threats.

Fleas (Order Siphonaptera) form a unique group of insects comprising 15 families with a total of about 220 genera and some 2,500 described species [5]. Five families including 25 genera are ectoparasites of birds, while all other flea species specifically feed on mammals. In Madagascar, located about 800 km west of Reunion Island, flea diversity has been extensively studied, mainly because of their role as vectors of *Yersinia pestis*, the plague agent, especially in this country that reports most human plague cases worldwide [6], [7]. Flea diversity is high in Madagascar, with several endemic species together with a few cosmopolitan ones, which host specificity and distribution have been partly described [6], [7]. Surprisingly, *Xenopsylla brasiliensis* (Baker, 1904) has never been collected in Madagascar, even though this species is recognized as a main plague vector in Eastern and Southern Africa [8], [9] and has been collected in Moroni (Grande Comore) and notified on two other islands of the Southwestern Indian ocean: Mayotte (Comoros archipelago) and Mauritius [10]. By contrast, almost no data are currently available on flea diversity on the other islands of the Southwestern Indian Ocean, including Reunion. The cosmopolitan and/or tropical species possibly present in the region are *Pulex irritans* Linné, 1758, *Echidnophaga gallinacea* Westwood, 1875, *Leptopsylla segnis* Schönherr, 1811, *Xenopsylla cheopis* Rothschild, 1903 as well as *Ctenocephalides spp.* Hence, the recent emergence of murine typhus in Reunion Island, where ten autochthonous human confirmed cases were reported between 2011 and 2013 (Balleydier E. 2014 pers. comm.) has stimulated the investigation of fleas for vector-assessment of indigenous species for the agent *Rickettsia typhi*. The objective of our study was therefore to report the diversity and distribution of fleas in Reunion Island with the aim of highlighting patterns of possible epidemiological importance.

## Materials and Methods

### Small mammals trapping

**Figure 1:**
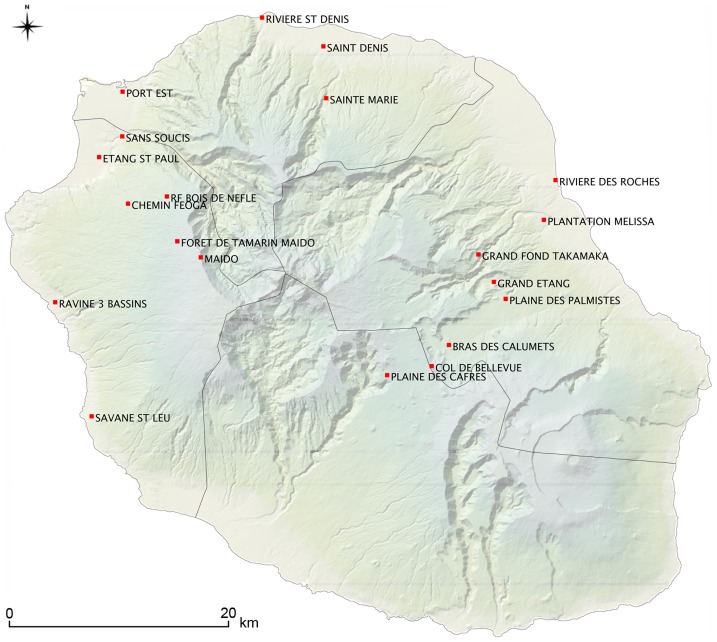
Sampling sites along the two altitudinal transects on western and eastern coasts, together with additional sampling sites in the north and west coast of Reunion Island.

Trapping was conducted throughout a one-year period survey (November 14^th^ 2012–November 16^th^ 2013) in different biotopes along two altitudinal transects lying on each side of the island: the eastern transect comprised eight sampling sites and the western transect, seven. In addition, two sites located in the western coast and a few sites in the urban northern part of the island were included in the present survey (Figure 1). The sampling encompassed the two local seasons, *i.e.* hot-wet summer from November to June, and cool-dry season from July to October, with twelve out of the twenty sampling sites being sampled twice, *i.e.* during the two seasons. Trapping was conducted following a standardized protocol: wire cage live traps (29 by 18 by 12 cm) were used for rats trapping (and accidentally tenrecs), and Sherman live traps for mice and shrews. On each sampling site, forty to eighty traps were placed in line approximately 15 meters apart in the afternoon; trapped animals were collected the following morning and brought back to the laboratory for processing. Traps were baited during three consecutive nights using successively within each line cheese, coconut or a mixture of peanut butter and canned sardine oil. This baiting setup (bait A, bait B, bait C, bait A, …) was implemented in order to trap most of the prevalent mammal diversity at each sampling site. Traps were left open in the same place during the day, with productive traps being immediately replaced every morning with the same bait over the 3-days trapping session.

### Ethics statement

Animals were sacrificed by cervical dislocation without anaesthesia to avoid bleeding in accordance with guidelines accepted by the scientific community for the handling of wild mammals [11] and the institutional guidelines published by the Centre National de la Recherche Scientifique (http://www.cnrs.fr/infoslabos/reglementation/euthanasie2.htm). All animal procedures carried out in this study were approved by the French Institutional Ethical Committee “Comité d'éthique du CYROI” (No. 114).

### Mammals and fleas' morphological diagnosis

Following sacrifice, each animal was visually examined for 10 minutes and all ectoparasites, including fleas, were manually collected either with a brush soaked in ethanol when insects jumped off the host, or forceps when eye-spotted in the fur. Collected fleas were preserved in 70% ethanol for later morphological and molecular analyses. Fleas were identified at the species level using taxonomic keys provided by Lewis [12], and Hoogstraal and Traub [13]. A subsample of Xenopsylla spp. fleas were mounted permanently on slides using Euparal medium, following a procedure adapted from Brigham Young University (http://fleasoftheworld.byu.edu/Systematics/MountingTechniques.aspx). The gender, genus, and species were recorded for each flea specimen. *Xenopsylla cheopis* and *X. brasiliensis* were mainly differentiated using the occurrence of marginal cones at the basis of the antepygidial bristle in males, and shape of spermatheca on mounted females [7]. Rodents body mass, ear, and back foot lengths, together with tail and body lengths were recorded. *Rattus* spp. was identified using morphological criteria including the comparison of (i) the ratio of tail to body lengths, (ii) the ear length and (iii) the hind foot length [14].

### Mammals and fleas' molecular diagnosis

The morphological diagnosis of *Rattus* spp. was confirmed by molecular data through sequencing of *cytB* locus from 15 randomly selected animals morphologically identified as *R. rattus* or *R. norvegicus*. Briefly, DNA was prepared from 20 mg of kidney tissue as previously described [15] and used as a template with L14723 and H15915 primers set, following a previously described PCR protocol [14].

For molecular diagnosis of fleas, DNA was prepared as follows: fleas were dried individually and subsequently crushed with a TissueLyser (Qiagen, Valencia, CA) using 3 mm tungsten beads and cetyl trimethyl ammonium bromide 2%; DNA was further extracted following a previously described procedure [16]. Both nuclear and mitochondrial loci were sequenced by amplifying 28S ribosomal RNA (28S rRNA gene) and cytochrome oxidase II (COII) encoding gene using 28S A/28S rD7b1 and COII F-leu/COII R-lys primer pairs, that produce 1473-bp and 770-bp PCR fragments respectively [17]. Amplicons were sequenced on both strands by Genoscreen (Lille, France) using the same PCR primers, and sequences were edited using Geneious Pro [18]. All sequences used in this study were deposited in Genbank and are accessible under accession numbers KJ638526 to KJ638590.

### Molecular analysis

All sequences were automatically aligned using MUSCLE implemented in Geneious Pro version 5.3.4 [18]. Alignments were constructed separately for the nuclear (28S) and mitochondrial (COII) datasets using sequences available in GenBank to complete our dataset. Bayesian analyses were performed to infer phylogenetic relationships between flea species. First, the best-fitting model and associated parameters were selected by jModelTest [19] and phylogenies were constructed by Bayesian inference. Two sets of four MCMCMC (Metropolis Coupled Markov Chain Monte Carlo) chains incrementally-heated were run in MrBayes 3.1.2 [20] for 20,000,000 generations. Trees and associated model parameters were sampled every 300 generations. The initial 2,000 trees were discarded as a conservative “burn-in” and the harmonic mean of the likelihood was calculated by combining the two independent runs. The 50% majority-rule consensus tree was then computed from the sampled trees in the two independent runs under the best model.

### Statistical analysis

The data were entered into EPIData 3.1 and analyzed with Epi info 6.04 statistical software using the chi-squared or Fisher exact tests for observed frequencies. We used a p-value threshold of 0.001. The effect of “habitat” on fleas' diversity was measured at two scales, host and sampling region, by using the flea percentage incidence index (PII: mammals parasitized by fleas of species A/mammals caught (%)), the specific flea index (SFI: number of fleas of species A collected from host species Y/mammals of species Y parasitized by fleas of species A) and the total flea index (TFI: total fleas collected/total trapped mammals, i.e. mean number of fleas per trapped mammal) [21]. The seasonality of flea diversity was tested by comparing PII on animals trapped at each site during the cool-dry versus hot-wet seasons.

## Results

### Flea sampling and morphological diagnosis

A total of 960 small mammals were trapped. They belong to the five introduced small terrestrial mammal species occurring in Reunion Island: 39 mice (*Mus musculus*), 168 shrews (*Suncus murinus*) and 25 tenrecs (*Tenrec eucaudatus*), all other specimens being rats (*Rattus rattus*: N = 554; *R. norvegicus*: N = 174) (Table 1). Almost 10% (95) of trapped mammals were infested with fleas (Table 1) and the TFI (mean number of fleas per host) was equal to 0.3 when based on all trapped mammals, and equal to 3 when based on parasitized mammals only. Of 288 fleas collected during the survey, 286 could be identified on a morphological basis. They were distributed within three genera and four distinct species, namely *Xenopsylla cheopis* (N = 171), *Xenopsylla brasiliensis* (N = 63), *Leptopsylla segnis* (N = 43) and *Echidnophaga gallinacea* (N = 9) (Table 2).

**Table 1:** Flea indices.

	PII No parasitized mammals/No mammals caught (%)				TFI
	North	East	West	Total	
Mice	-	5/11 (45.5)	0/28 (0)	5/39 (12.8)	20/39 (0.5)
*RN*	6/19 (31.6)	1/90 (1.1)	3/65 (4.6)	70/174 (5.7)	33/174 (0.2)
*RR*	1/26 (3.8)	1/291 (0.3)	69/237 (29.1)	71/554 (12.8)	221/554 (0.4)
Shrews	2/27 (7.4)	0/72 (0)	6/69 (8.7)	8/168 (4.8)	12/168 (0.1)
Tanrecs	-	1/19 (5.3)	0/6 (0)	1/25 (4.0)	2/25 (0.1)
Total	9/72 (12.5)	8/483 (1.7)	78/405 (19.3)	95/960 (9.9)	288/960 (0.3)

Sample results by host species and region indicating the number of mammals parasitized by fleas per total number of trapped mammals (PII index in brackets), and the mean number of fleas per trapped mammal (TFI index in brackets). *RN: Rattus norvegicus; RR: Rattus rattus*.

**Table 2:** Number of collected fleas per flea and host species.

	*X. cheopis*	*X. brasiliensis*	*L. segnis*	*E. gallinacea*	NA	Total
Mice	0	0	19	0	1	20
*RN*	32	0	1	0	0	33
*RR*	126	62	23	9	1	221
Shrews	11	0	0	0	0	12
Tenrecs	2	1	0	0	0	2
Total	171	63	43	9	2	288

NA: non available (unidentified escaped fleas).

### Host prevalence and host specificity

*Rattus rattus* was found to be the most parasitized host, predominantly by *Xenopsylla* spp. (*p*<10^−3^). Only five mice, eight shrews and one tenrec were found parasitized by fleas (*Xenopsylla* spp. and *L. segnis*) (Table 2). *Rattus rattus* was more heavily infested in the western side of the island (*p*<10^−7^) whereas *R. norvegicus* was most infested in the northern part (*p*<10^−4^) and mice in the eastern part (*p*<10^−4^). No difference according to the sampling region was found in shrews or tenrecs. When considering *Xenospylla* spp., *X. cheopis* was mainly found on *Rattus spp.* (*p*<10^−5^) with no difference between *R. rattus* and *R. norvegicus* but *X. brasiliensis* was significantly more abundant on *R. rattus* (*p*<10^−4^) than on any other mammal species. The number of flea species per host species ranged from one to four (Table 2), but most mammals were parasitized by a single flea species although nine *R. rattus* were found co-infested with two distinct species as follows: *X. cheopis*+*X. brasiliensis* (N = 1), *X. cheopis*+*E. gallinacea* (N = 3), and *X. cheopis*+*L. segnis* (N = 3).

### Flea index and distribution

**Figure 2:**
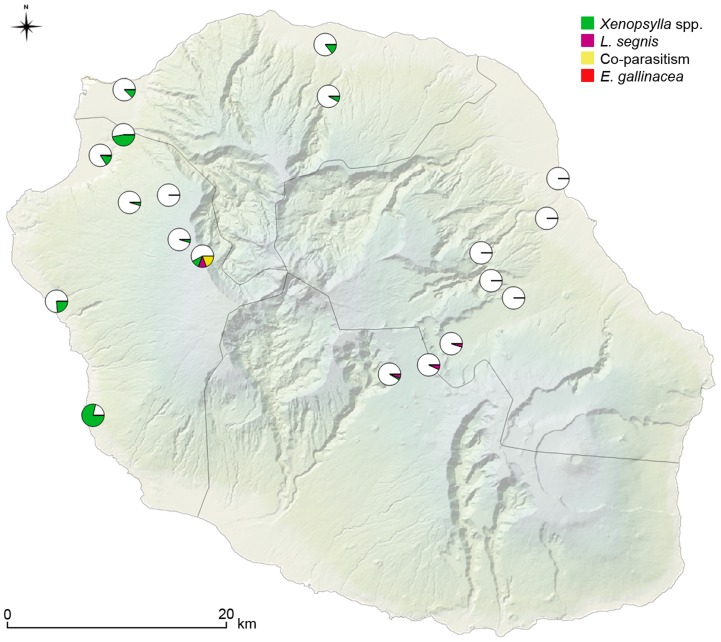
Prevalence of flea infestation on mammal hosts, *i.e.* percentage of hosts infested by different flea species over all captured hosts, for each sampling site.

*Xenopsylla* spp. were by far the most common fleas (234/286 fleas) with *X. cheopis* and *X. brasiliensis* representing 59% (171/286) and 22% (63/286) of all identified fleas, respectively (Table 2). *Xenopsylla cheopis* was also the most geographically widespread species, as it was present in all of the fourteen flea-positive sampling sites out of the twenty prospected ones. *X. brasiliensis* was collected at only two sites throughout the island, both of them being located on the western transect. Noteworthy, *X. brasiliensis*/*R. rattus* SFI index was relatively high in one of those 2 sites (Sans Soucis, SFI = 2). *Leptopsylla segnis* was collected on mice and both rat species in four elevated sites (>1,000 meters), and *E. gallinacea* was only collected on *R. rattus* at three distinct sites along the western transect (Figure 2).

Windward and leeward transects displayed dramatically different results, in terms of abundance of fleas and species richness (Table 3). The PII was significantly lower (*p*<10^−7^) in the eastern region compared to the northern and western regions. Indeed, 201 *Xenopsylla* fleas were collected out of 405 mammals trapped in the western transect while this species was totally absent on the 464 rodents trapped on the eastern transect (see Tables 1, 3); the only two *X. cheopis* specimens collected in the eastern side were from one tenrec trapped on the top of the eastern transect located in an elevated plateau at the centre of the island (Table 3; Figure 2). All other fleas collected in the eastern transect were identified as *L. segnis* (21 of 24 collected fleas; Table 3).

**Table 3:** Number of collected fleas per flea species in each region.

	North	East	West	Total
*X. cheopis*	31	2	139	171
*X. brasiliensis*	0	0	62	63
*L. segnis*	0	21	22	43
*E. gallinacea*	0	0	9	9
NA	0	1	1	2
Total	31	24	233	288

NA: non available (unidentified escaped fleas).

Lower flea species richness was recorded in animals trapped along the eastern than in the western transect: fleas were absent on six of the nine eastern sampling sites,and on the remaining sites, only seven mammals were found parasitized. The specific flea indexes (SFI) were 1.47 for *X. cheopis/R. norvegicus* on the northern sampling sites; 0.53 for *X. cheopis/R. rattus* in the western sites; and 0.26 for *X. brasiliensis*/*R. rattus* in the western sites (see Tables 4 and 5).

**Table 4:** Indices of *X. cheopis* fleas according to small mammal host species trapped during the survey.

	PII *X. cheopis*				SFI *X. cheopis*
	North	East	West	Global	
Mice	-	0/11 (0)	0/28 (0)	0/39 (0)	0 (0/39)
*RN*	6/19 (31.6)	0/90 (0)	3/65 (4.6)	9/174 (5.2)	0.2 (32/174)
*RR*	0/26 (0)	0/291 (0)	40/237 (16.9)	40/554 (7.1)	0.2 (126/554)
Shrews	2/27 (7.4)	0/72 (0)	5/69 (7.2)	7/168 (4.2)	0.1 (11/168)
Tenrecs	-	1/19 (5.3)	0/6 (0)	1/25 (4.0)	0.1 (2/25)
Total	8/72 (11.1)	1/483 (0.2)	48/405 (11.9)	57/960 (5.9)	0.2 (171/960)

PII: percentage incidence index; PII *X. cheopis*: mammals parasitized by *X. cheopis*/mammals caught (%); TFI: total flea index; SFI: specific flea index.

**Table 5:** Indices of *X. brasiliensis* fleas according to small mammal host species trapped during the survey.

	PII *X. brasiliensis*				SFI *X. brasiliensis*
	North	East	West	Global	
Mice	-	0/11 (0)	0/28 (0)	0/39 (0)	0 (0/39)
*RN*	0/19 (0)	0/90 (0)	0/65 (0)	0/174 (0)	0 (0/174)
*RR*	0/26 (0)	0/291 (0)	26/237 (11.0)	26/554 (4.7)	0.1 (62/554)
Shrews	0/27 (0)	0/72 (0)	1/69 (1.4)	1/168 (0.6)	0 (1/168)
Tanrecs	-	0/19 (0)	0/6 (0)	0/25 (0)	0 (0/25)
Total	0/72 (0)	0/483 (0)	27/405 (6.7)	27/960 (2.8)	0.1 (63/960)

PII *X. brasiliensis*: percentage incidence index: mammals parasitized by *X. brasiliensis*/mammals caught (%).

### Seasonality of infestation

There is no apparent seasonality of flea abundance in the eastern region, which could be explained by the absence or very low abundance of fleas, even during the peak season observed on other parts of the Island. Seasonality is observed in the west, with greater abundance observed during the hot-wet season. Over the fourteen flea-positive sampling sites, seven were sampled during the two seasons. Two sampling sites were flea-positive during both seasons, four were flea-positive only during the hot-wet season and one was found flea-negative during the hot-wet season, and flea-positive during the cool-dry season (one *R. norvegicus* and one *S. murinus* parasitized by one *X. cheopis* flea each), but the difference was not statistically significant (Table 6). This seasonality was significant for *X. brasiliensis* on sampling site « Sans soucis » (*p* = 0.01; RR = 2.2 [1.1–4.3]), and for *X. cheopis* on sampling site « Port est » (*p*<10^−3^; RR = 11.7 [1.6–86.5]).

**Table 6:** Flea indices and seasons.

	Sample site	PII (%)		TFI total*		TFI parasitized*		Flea species
		Cool-dry season	Hot-wet season	Cool-dry season	Hot-wet season	Cool-dry season	Hot-wet season	
NORTH	Ravine du chaudron	0/4 (0)	1/5 (20.0)	0	7/5 (1.4)	0	7/1 (7)	*X. cheopis*
EAST	Gd Fond Takamaka	0/34 (0)	1/21 (4.8)	0	1/21 (0.05)	0	1/1 (1)	(1 escaped flea)
	Col de Bellevue	1/11 (9.1)	1/19 (5.3)	1/11 (0.1)	2/19 (0.1)	1/1 (1)	2/1 (2)	*L. segnis*
WEST	Port est	0/44 (0)	12/45 (26.7)	0	17/45 (0.4)	0	17/12 (1.4)	*X. cheopis* (1 escaped flea)
	Sans Soucis	7/25 (28.0)	17/26 (65.4)	11/25 (0.4)	50/26 (1.9)	11/7 (1.6)	50/17 (2.9)	*X. brasiliensis*
	Feoga	0/17 (0)	2/27 (7.4)	0	2/27 (0.1)	0	2/2 (1)	*X. brasiliensis*
	Forêt tamarins Maïdo	2/19 (10.5)	0/27 (0)	2/19 (0.1)	0	2/2 (1)	0	*X. cheopis*

Indices of flea infestation for each of the 7 flea-positive sampling sites (out of 12) where mammals were collected twice. Results are presented for each season (cool-dry vs. hot-wet). The five flea-negative sampling sites are not presented. TFI total: mean number of fleas by trapped mammal; TFI parasitized: mean number of fleas by infested mammal.

### Molecular analysis

**Figure 3:**
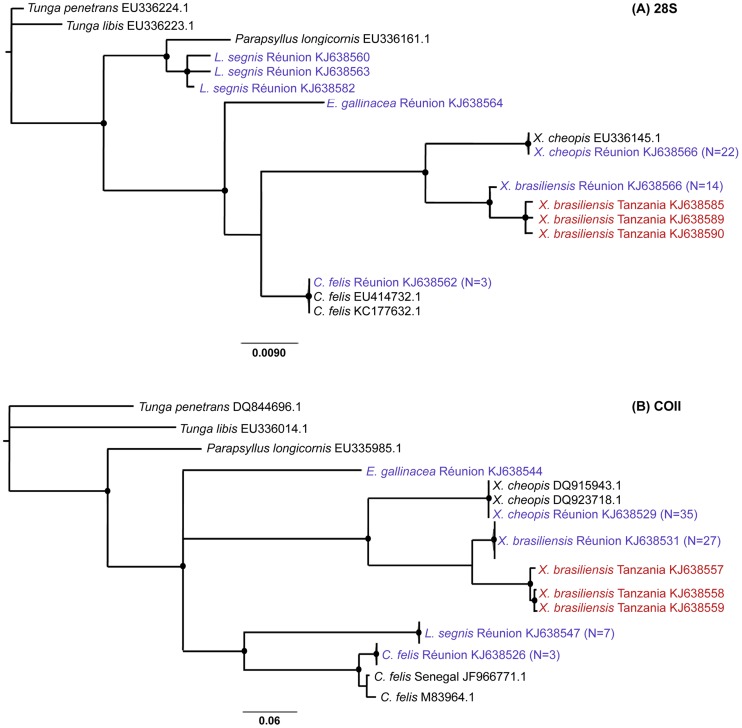
Flea phylogenetic trees constructed with (A) nuclear 28S and (B) mitochondrial COII markers. Sequences obtained in the present study are coloured (red for Tanzania, purple for Reunion Island). Only bootstrap supports >70% are shown (black dots). Genbank accession numbers are indicated. When several sequences obtained from different fleas showed 100% sequence identity, only one of them was written with the number (N) of identical haplotypes between brackets. Sequences obtained from three cat fleas (*Ctenocephalides felis*) sampled in the house of a murine thyphus human case were included in the analysis.

Sixty (28S) and seventy (COII) sequences were obtained from fleas sampled in Reunion Island. As all sequences of *X. cheopis* and *X. brasiliensis* were 100% identical, only a dozen sequences representative of each of those two species were included in the analyses. Few sequences from Genbank were added, including *Parapsyllus longicornis* used as an extra-group. Since no 28S or COII sequences were available on databases for *X. brasiliensis*, we sequenced three *X. brasiliensis* specimens sampled in Tanzania (KJ638557-59 in COII; KJ638585, 638589-90 in 28S: collectors Laudisoit A., Makundi R., Katakweba A., S3°58′989″ E35°21′560″, 1994 m, 10/02/2009). Models selected by jModelTest were GTR+I for 28S phylogeny (AIC weight = 0.62), and GTR+G for COII phylogeny (AIC weight = 0.85). All *X. cheopis* (from Reunion Island and two haplotypes from Genbank, 28S sequence) branched within a single well supported clade, while *X. brasiliensis* haplotypes fell within two well supported clades, one containing sequences from Tanzanian fleas, the second harboring all haplotypes from Reunion Island (Figure 3). Both clades formed a well supported monophyletic *X. brasiliensis* clade distinct from *X. cheopis* and embedded within *Xenopsylla* group.

## Discussion

The present investigation provides the first information on flea diversity and distribution on the five introduced small mammal species present on Reunion Island, where no data were available thus far. We describe the presence of three genera composed of four distinct cosmopolitan species, namely *X. cheopis*, *X. brasiliensis*, *L. segnis* and *E. gallinacea*. Morphological diagnosis of *X. cheopis* and *X. brasiliensis* was further confirmed by sequencing of 28S and COII markers: for *X. cheopis*, fleas sampled in Reunion Island showed 99% and 100% identity with sequences accessible in Genbank (*i.e.* EU336145.1 and HM188404.1 for 28S and COII sequences, respectively). As no sequences were currently available for *X. brasiliensis* on these 2 loci, we generated sequence data using specimens previously sampled in Tanzania and morphologically identified as *X. brasiliensis* by A. Laudisoit and colleagues. Again, molecular data confirmed *X. brasiliensis* morphological diagnosis, with 28S and COII sequences obtained from fleas sampled in Reunion Island showing respectively 99% and 94% identity with sequences obtained from Tanzanian fleas.

Phylogenetic analysis carried out with both nuclear and cytoplasmic markers provided two well resolved mostly congruent trees, suggesting that no hybridization nor introgression (two molecular events known to lead to molecular misdiagnosis [22]) has occurred within our sample. However, the analyses did reveal one incongruency for *L. segnis*: while 28S-based analysis was coherent with classical taxonomy, COII sequences unexpectedly clusterized *L. segnis* within Pulicidae. Additional and more informative markers need to be investigated in order to address this incoherence together with other more basic questions such as a previously reported paraphylly of Leptopsyllidae [23]. The absence of molecular data for *L. segnis* together with the overall scarcity of accessible DNA sequences for other flea species (including *X. brasiliensis*, see above) should stimulate an increased effort towards the release of a proper barcoding tool facilitating the diagnosis of cosmopolitan species. As for *X. brasiliensis*, nuclear and mitochondrial sequences from Tanzanian specimens formed a cluster separated from Reunion Island sequences, which might indicate an ongoing diversification. However, a proper investigation of eastern African and Indian Ocean *X. brasiliensis* populations would be required to ascertain any level of genetic structuration. Altogether, our data indicate a low diversity of fleas on small mammals from Reunion Island. In addition, all flea species were cosmopolitan and likely result from the recent introduction of their vertebrate hosts on the island, or from the importation of food stocks with preimaginal stages. This feature is not unexpected considering the low specific richness in mammal hosts, which strikingly contrasts with the neighbouring island of Madagascar where species richness and endemism of both flea [7] and small mammal hosts are high [24], flea endemism likely resulting from long host-parasite co-evolutionary processes.

Host specificity differed between fleas: *E. gallinacea* was only collected on *R. rattus* which is likely a spill over host from poultry breeding sites near the concerned sampling sites, *i.e.* rural areas where *R. norvegicus* is likely to be less common. *Xenopsylla brasiliensis* appeared mostly associated with *R. rattus* (one flea found on a shrew) a situation reminiscent to that previously described in the Canary islands [25]. On the contrary there was low host specificity for *X. cheopis* that was found to most commonly infest *Rattus* spp. (92%), but was also found on shrews and tenrecs (Tables 4 and 5), which is in accordance with previous report from Madagascar [7]. The number of collected specimens from the two other species was too low to conclude about host specificity.

This is the first report of *Xenopsylla brasiliensis* in Reunion Island. This species is native to continental subsaharian Africa where it is the most common plague vector in some areas, often more abundant than *X. cheopis* [9]. This expanding species has spread to other parts of the world such as Brazil and India [26]. This known plague vector, particularly effective in rural environments, is less tolerant to high temperatures than *X. cheopis* but is more resistant to drier conditions [21]. These ecological traits are in agreement with *X. brasiliensis* distribution in Reunion Island, where the species was restricted -in our sample- to a semi-xerophil landscape partly covered with *Tamarinus indica* and patches of exotic *Furcraea foetida* and *Agave americana* on the western side of the island.

The heterogeneous distribution of fleas over Reunion Island, with no *Xenopsylla* flea collected along the windward humid eastern side, might be related to excessive rainfall in this coast. Indeed, temperature, rainfall and relative humidity have direct effects on development and survival of fleas, and a direct effect of rainfall is supposed to occur when high intensity rainfall causes flooding of rodent burrows [27]. Seasonal abundance of fleas that has been largely reported in literature is also driven by climate variables. Warm-moist weather has been described to provide higher flea indices [27]. This is in agreement with the decrease in flea abundance observed during the cool-dry season on the two sampling sites were seasonality was significant.

Fleas are of tremendous medical and economic importance as vectors of several diseases including bubonic plague, murine typhus and tularaemia [28]. The discovery of fleas as vectors of *Yersina pestis*, and later of *Rickettsia typhi*, the ethiological agent of murine typhus, stimulated flea studies in the early 20^th^ century. *Xenopsylla cheopis* is now considered as the most important cosmopolitan vector of both *Y. pestis* and *R. typhi*, and an important *Bartonella* spp. carrier, and *X. brasiliensis* is an efficient plague vector, especially in rural environments. *Leptopsylla segnis* is a weak vector of *Y. pestis* according to old standards (but no recent experimental studies have been performed to establish if the early-phase transmission apply to this species) and is a dubious vector of *R. typhi* [29]. Hence, our study showing that Reunion Island hosts several flea species of medical importance warrants better surveillance of potentially emerging flea-borne zoonoses.

Among flea-borne diseases, the situation of plague is of major concern for the region. Plague was introduced in Madagascar from India in 1898 and has become endemic in the highlands [30]. *Xenopsylla cheopis* and the endemic flea *Synopsyllus fonquerniei* are known as the primary vectors of *Y. pestis* on Madagascar [31]. In Reunion Island, plague has quite a long history: the disease was likely misdiagnosed as lymphatic filariasis until 1899 when *Y. pestis* was isolated by André Thiroux and formally identified by Emile Roux [32]. Thus plague was described within the same year in Madagascar, Reunion and Hawaii, but it was considered as introduced in Madagascar [32] and Hawaii [33] where foci were first described in harbors, while André Thirioux described plague as endemic in Reunion [32].

Plague is not a concern anymore in Reunion Island where the last human cases were reported in 1926 [34]. Indeed, an SFI of 0.5 to 1 is considered sufficient to maintain plague in a locality and an index ≥1 is reported to represent a potentially dangerous situation with respect to the risk of plague outbreak [8]. Some indexes reported herein (Tables 4 and 5), specifically the *X. cheopis/R. norvegicus* SFI measured on the north of the island may be considered of concern and should be monitored systematically. This area is close to the city of Le Port, the only international harbour of Reunion Island, and the most likely entry port for parasitized rodents and/or food. Although the risk of plague introduction from Madagascar is expected to be limited with an SFI index in this area <0.5 [7], the substantial shipping trade between Reunion and Madagascar where plague has already been described in harbours [35], [36] command a cautious control in order to prevent introduction of rodents from this plague endemic country [28]–[29]. Finally, the role of domestic cats should not be overlooked since Felidea – in contrast to Canidea in general - are sensitive to the disease, can become infected by ingesting infested rodents and develop pulmonary form of the disease, with a risk of direct respiratory transmission of infectious droplets to the people caring for them [37].

Considering other flea-borne diseases, rickettsioses represent an important concern. Interestingly, a retrospective French study (2008–2010) on travellers returning from Madagascar and Reunion reported two patients who were infected with murine typhus during their trip [38]. More recently, in 2012 and 2013, several autochthonous human confirmed cases of murine typhus were reported by hospital clinicians from the western and southern parts of the island (Balleydier E., pers. comm.). The authors were wondering if the heterogeneous distribution of human cases could be related to medical surveillance bias. Although incomplete, since the southern coast of the island wasn't sampled, the distribution of fleas reported herein is at least in part overlaid with that of human cases. This may suggest that the risk of murine typhus in Reunion Island is related to fleas' geographical distribution driven by environmental determinants. The detection of *R. typhi* in fleas together with the presentation of a more complete *Xenopsylla* sp. distribution map throughout the island may provide public health agencies with a useful tool for implementing a specific surveillance system for better risk assessment of murine typhus and other emerging flea-borne zoonoses in Reunion Island.

## Supporting Information

Table S1 Geographical and environmental information on sampling sites. Corine codes together with habitat description correspond to those listed in the CORINE Biotope Réunion Database (http://www.reunion.developpement-durable.gouv.fr/typologie-corine-biotope-reunion-a158.html).
(DOC)
