# Correction: Fleas of Small Mammals on Reunion Island: Diversity, Distribution and Epidemiological Consequences

**DOI:** 10.1371/journal.pntd.0003264

**Published:** 2014-09-19

**Authors:** The PLOS Neglected Tropical Diseases Staff

There is a missing affiliation for the 8th author. Koussay Dellagi is affiliated with: Centre de Recherche et de Veille sur les Maladies Emergentes dans l’Océan Indien, Plateforme de Recherche CYROI, Sainte Clotilde, Reunion Island, France, and Institut de Recherche pour le Développement, Sainte Clotilde, Reunion Island, France.
