# Supplementary material for: Fleas of Small Mammals on Reunion Island: Diversity, Distribution and Epidemiological Consequences
Source: PLoS Negl Trop Dis. 2014 Sep 4;8(9):e3129. doi: 10.1371/journal.pntd.0003129 (PMC4154673; doi:10.1371/journal.pntd.0003129)
Supplement: Table S1 — Geographical and environmental information on sampling sites. Corine codes together with habitat description correspond to those listed in the CORINE Biotope Réunion Database (http://www.reunion.developpement-durable.gouv.fr/typologie-corine-biotope-reunion-a158.html). (DOC) [file pntd.0003129.s001.doc]

| **SITE** | **LONGITUDE (WGS84 UTM South 40)** | **LATITUDE (WGS84 UTM South 40)** | **ALTITUDE (meters)** | **ALTITUDE CATEGORY** | **SECTOR** | **COAST** | **VEGETATION STAGE** | **CLIMATE** | **LAND USE** | **HABITAT DESCRIPTION (ACCORDING TO CORINE BIOTOPE REUNION DATABASE)** | **CORINE CODE** | **WATER PROXIMITY** | **NATURALNESS** |
| --- | --- | --- | --- | --- | --- | --- | --- | --- | --- | --- | --- | --- | --- |
| **BRAS DES CALUMETS** | 355261.159528 | 7660796.933768 | 1160 | High | Eastern transect | Winward | Nepheliphile | Mesotherm | Farming area next degraded native forest | Mountain shrub of *Erica reunionensis,*  grass sown | 39.4113 and 81.00 | Gully | Medium |
| **CHEMIN FEOGA** | 325892.837329 | 7673728.938734 | 840 | Medium | Western transect | Leeward | Hygrophile | Megatherm | Farming area | Secondary formation of *Acacia mearnsii*  grass sown | 87.1956 and 81.00 | no | Low |
| **COL DE BELLEVUE** | 353684.409265 | 7658862.973016 | 1610 | High | Eastern transect | Winward | Nepheliphile | Mesotherm | Picnic area and walking trails in primary forest | Mountain shrub of *Erica reunionensis* | 39.4113 | no | High |
| **ETANG ST PAUL** | 323250.700438 | 7677989.374993 | 10 | Low | Western transect | Leeward | Hygrophile | Megatherm | Peri urban and farming areas | Swamp vegetation of low altitude, savannah, ruderal wasteland of *Panicum maximum*,industrial wasteland | 59.211 and 97.1922 and 87.1912 and 86.40 | Wetland | High |
| **FORET TAMARINS MAIDO** | 330406.489301 | 7670290.666433 | 1770 | High | Western transect | Leeward | Nepheliphile | Mesotherm | Picnic area and walking trails in secondary forest | Forest of *Accacia heterophylla* and *Nastus borbonicus* and cultivated forest of  *Tamarindus indica* | 49.3144 and 83.392 | Gully | Medium |
| **GRAND ETANG** | 359376.971031 | 7666566.058749 | 540 | Medium | Eastern transect | Winward | Hygrophile | Megatherm | Lake, walking trails in secondary forest | Swamp vegetation of medium altitude, secondary shrub of *Psidium cattleianum* | 59.212 and 87.1955 | Lake | High |
| **GRAND FOND TAKAMAKA** | 357960.801497 | 7669082.895913 | 655 | Medium | Eastern transect | Winward | Hygrophile | Megatherm | Picnic area and walking trails in primary forest | Hygrophile forest of medium altitude | 49.112 | Wetland | High |
| **MAÏDO** | 332551.007548 | 7668815.464365 | 2215 | High | Western transect | Leeward | Heath | Oligotherm | Walking trails | Heath, mattorals, and shrub of high altitude, secondary shrub of *Ulex europaeus* | 39.42 and 87.1957 | Gully | High |
| **PLAINE DES CAFRES** | 349610.599444 | 7658035.689137 | 1610 | High | Eastern transect | Winward | Nepheliphile | Mesotherm | Farming area | Grass sown | 81.00 | no | Low |
| **PLAINE DES PALMISTES** | 360455.155134 | 7665018.811083 | 790 | Medium | Eastern transect | Winward | Hygrophile | Megatherm | Walking trails in primary vegetation | Hyperhumid mountain shrub of *Pandanus montanus* | 39.42 | Wetland | High |
| **PLANTATION MELISSA** | 363947.893639 | 7672248.833444 | 75 | Low | Eastern transect | Winward | Hygrophile | Megatherm | Orchard | Orchard (Letchi) | 83.10 | River | Medium |
| **PORT EST** | 325397.586366 | 7683976.620788 | 25 | Low | Western transect | Leeward | Semi-xerophile | Megatherm | Main island harbor, container terminal | Shrub savannah of *Pithecllobium dulce* and *Albizia lebbeck,* seaport | 87.1921 and 89.11 | Gully | Null |
| **RAVINE 3 BASSINS** | 319233.161665 | 7664709.798328 | 25 | Low | Western transect | Leeward | Semi-xerophile | Megatherm | Secondary savannah | Savannah of *Pithecllobium dulce* and *Albizia lebbeck* | 87.1921 | Pond | High |
| **RAVINE DU CHAUDRON** | 343773.686517 | 7688124.534309 | 55 | Low | North | Winward | Hygrophile | Megatherm | Urban | Gully in urban area |  | Gully | Low |
| **RF BOIS DE NEFLES** | 329453.486983 | 7674387.966982 | 1230 | High | Western transect | Leeward | Nepheliphile | Mesotherm | Farming area next primary forest | Hygrophile mountain forest of *Dombeya spp.*, grass sown | 49.3126 and 81.00 | no | High |
| **RIVIÈRE DES ROCHES** | 365016.399127 | 7675883.383211 | 10 | Low | Eastern transect | Winward | Hygrophile | Megatherm | Walking trails in coastal peri-urban area | Shrub beaches, humid ruderal fallow | 17.912 and 87.1912 and 83.397 | Wetland | Low |
| **RIVIERE ST DENIS** | 338163.081244 | 7690754.657368 | 10 | Low | North | Winward | Hygrophile | Megatherm | Urban | Riverside in urban area |  | River | Low |
| **SAINT DENIS** | 341137.519732 | 7685549.363340 | 440 | Low | North | Winward | Hygrophile | Megatherm | Urban | Village | 86.20 | no | Null |
| **SAINTE MARIE** | 344034.523227 | 7683377.935459 | 255 | Low | North | Winward | Hygrophile | Megatherm | Farming area and quarry | Traditional culture, quarry | 82.30 and 86.41 | River | Medium |
| **SANS SOUCIS** | 325366.628154 | 7679885.595596 | 150 | Low | Western transect | Leeward | Semi-xerophile | Megatherm | Farming area next secondary vegetation | Shrub savannah of *Leucaena leucocephala*, *Listea glutinosa,* *Albizia lebbeck* and *Furcrea foetida*. Forest of *Tamarindus indica* | 87.1934 and 87.1922 and 87.1943 | no | Medium |
| **SAVANE ST LEU** | 322568.943825 | 7654273.670737 | 90 | Low | West | Leeward | Semi-xerophile | Megatherm | Picnic area in secondary savannah and peri-urban area | Shrub savannah of *Leucaena leucocephala,*  Village | 87.1933 and  86.20 | no | Medium |

**Table S1**
